# Supplementary material for: Coming together in a digital age: Community twitter responses in the wake of a campus shooting
Source: PLoS One. 2022 Dec 28;17(12):e0279569. doi: 10.1371/journal.pone.0279569 (PMC9797086; doi:10.1371/journal.pone.0279569)
Supplement: S1 Table — (DOCX) [file pone.0279569.s001.docx]

| **Emotion Label** | **Description** |
| --- | --- |
| ***Negative Emotions*** |  |
| Anger/Aggression | Hate |
| Sadness/Grief | Crying emoji; Mourning; “Loss” |
| Anxiety/Worry | Anxious; Concerns about the future or what will come; “Afraid to go back to campus” |
| Fear | General fear; NOT related to future |
| Uncertainty/Confusion | Lack of knowing |
| Disbelief/Shock | Can’t believe; Unreal |
| ***Positive Emotions*** |  |
| Humor | Sarcasm; Crying while laughing emoji |
| Hope | Positive ideas about the future |
| Appreciation | Appreciation of victims; Gratitude |
| ***Communal Response*** |  |
| Thoughts/Prayers | Extending thoughts and/or prayers |
| Request/Need Support | Any request/need for support for self or others - any form |
| Healing/Community Response | #NinerStrong; vigil; |
| Victim Remembrance/ Honor | Mentions of Riley Howell, Ellis "Reed" Parlier, Drew Pescaro, Sean DeHart, Rami Al-Ramadhan, Emily Houpt |
| Blame/Responsibility | Blame of entities or individuals for gun violence; Not just on people; “Fault” “blame” “on your hands”; “This is what happens when you…” |
| ***Action*** |  |
| Action Taken | If author said they took an action |
| Policy Advocacy | Policies of organizations, campus, national policy/law |
| Individual Advocacy | Advocating for victims or individuals - NOT larger change |
| ***Information*** |  |
| Death/Injury | Reports of deaths/injuries; descriptions of deaths or injuries. |
| Official Response | Messages from government, campus (Dean, Chancellor, Campus police), city police |
| Warning Message | Warning others about shooting |
| Reaction to Media | Response to news story - NOT social media reactions specifically |
| News | Any news source or article |
