# Supplementary material for: Coming together in a digital age: Community twitter responses in the wake of a campus shooting
Source: PLoS One. 2022 Dec 28;17(12):e0279569. doi: 10.1371/journal.pone.0279569 (PMC9797086; doi:10.1371/journal.pone.0279569)
Supplement: S2 Table — (DOCX) [file pone.0279569.s002.docx]

| **Emotion Label** | **Fleiss’ Kappa** |
| --- | --- |
| ***Negative Emotions*** |  |
| Anger/Aggression | 0.598 |
| Sadness/Grief | 0.584 |
| Anxiety/Worry | -0.007 |
| Fear | 0.358 |
| Uncertainty/Confusion | 0.277 |
| Disbelief/Shock | 0.321 |
| ***Positive Emotions*** |  |
| Humor | 1.0 |
| Hope | 0.373 |
| Appreciation | 0.625 |
| ***Communal Response*** |  |
| Thoughts/Prayers | 0.757 |
| Request/Need Support | 0.195 |
| Healing/Community Response | 0.764 |
| Victim Remembrance/ Honor | 0.796 |
| Blame/Responsibility | 0.563 |
| ***Action*** |  |
| Action Taken | 0.313 |
| Policy Advocacy | 0.518 |
| Individual Advocacy | -0.178 |
| ***Information*** |  |
| Death/Injury | 0.332 |
| Official Response | 0.249 |
| Warning Message | -0.007 |
| Reaction to Media | 0.420 |
| News | 0.530 |
