# Supplementary material for: Coming together in a digital age: Community twitter responses in the wake of a campus shooting
Source: PLoS One. 2022 Dec 28;17(12):e0279569. doi: 10.1371/journal.pone.0279569 (PMC9797086; doi:10.1371/journal.pone.0279569)
Supplement: Attachment — Submitted filename: Response to reviewers.docx [file pone.0279569.s003.docx]

**When submitting your revision, we need you to address these additional requirements.**

**1. Please ensure that your manuscript meets PLOS ONE's style requirements, including those for file naming. The PLOS ONE style templates can be found at**

[**https://journals.plos.org/plosone/s/file?id=wjVg/PLOSOne_formatting_sample_main_body.pdf**](https://journals.plos.org/plosone/s/file?id=wjVg/PLOSOne_formatting_sample_main_body.pdf)  **and**

[**https://journals.plos.org/plosone/s/file?id=ba62/PLOSOne_formatting_sample_title_authors_affiliations.pdf**](https://journals.plos.org/plosone/s/file?id=ba62/PLOSOne_formatting_sample_title_authors_affiliations.pdf)

We have edited the formatting as requested.

**2. You indicated that ethical approval was not necessary for your study. Could you please provide further details on why your study is exempt from the need for approval and confirmation from your institutional review board or research ethics committee (e.g., in the form of a letter or email correspondence) that ethics review was not necessary for this study? Please include a copy of the correspondence as an ""Other"" file.**

Twitter data are publicly available, and therefore not human subjects research as per our institutional review board. We have uploaded the correspondence as requested.

**Reviewer #1: The paper presents the result of collecting tweets related to
the 2019 UNC Charlotte shooting.
A large number of tweets were first filtered and the resulting subset
then annotated manually by several annotators. As such the paper
describes significant work that can be useful for a variety of purposes
e.g. to do further analysis or for use as a resource for NLP.**

We appreciate the reviewer's acknowledging the variety of applications for this work. **However the paper is very vague about many important aspects: what exactly
where the research questions that this works seeks to answer? How exactly
where the annotation guidelines developed and how do they deal with
edge cases, ambiguity, cases not considered in advance?
From the description in the paper it is not clear to me which categories
exactly could get assigned to each tweet (e.g. was it possible to assign
more than one or zero negative emotions to a tweet? Was it possible to
remain undecided? Was at least one label assigned to each tweet for each
of the groups "negative emotions", "positive emotions", "communal response",
"action" etc? The paper mentions in passing that labels from mroe than one
of the groups can be assigned, but there is no precise explanation.**

We appreciate the reviewer's request for more detail on our processes. On page 6, line 132 we have added in the following research questions: "For these reasons, we sought to answer the following research question: 1) What is the nature of non-affective short-term reactions to the UNC Charlotte campus shooting?; 1a) How do the reactions fluctuate in the week following the shooting?." We have added more information about the development of the codebook, and how it was augmented after initial analyses. We have added in the following to clarify how coders addressed each tweet: "Each tweet could receive multiple codes, no codes (this did not occur in our sample as non-relevant codes were discarded beforehand). Coders were instructed to contact the team for questionable tweets, which were discussed in weekly meetings during the coding period." (p8)

**What exactly was the allocation strategy for multiple annotations per
tweet? How many tweets were annotated by each annotator?**

We have added in information about the number in each of the coders' subsamples, which were randomly selected. "Each of the student coders coded a random subsample of approximately 3,600 tweets and the PI coded 1,953 tweets." (p8 L184).

**There is no proper analysus of inter annotator agreement,
and it is hard to figure out what should have been added given the poor
detail on the annotations and annotation process itself. However it is likely
that nominally scale Krippendorff alpha would probably have been a good
metric to look at. The paper also does not mention how agreement metrics
change e.g. when looking only at presence/absence of negative emotions rather
then detailed negative emotion labels.**

We approached this analysis from a qualitatively driven perspective. Computations of inter-rater agreement are debated among qualitative researchers (e.g. O'Connor & Joffe, 2020; Morse, 2018). Statistical approaches, though many quantitatively-focused researchers rely heavily on them, may have drawbacks (such as how they deal with chance agreement or difficulty in interpretation; O'Connor & Joffe, 2020). Rather than relying on the statistics of the inter-rater reliability, we emphasized our group discussion and understanding which is reflected in the codebook. To improve transparency, we have added our preliminary codebook as Appendix A.

O’Connor, C., & Joffe, H. (2020). Intercoder reliability in qualitative research: debates and practical guidelines. International journal of qualitative methods, 19, 1609406919899220.

Morse, J. M. (2018). Reframing rigor in qualitative inquiry. In Denzin, N. K., Lincoln, Y. S. (Eds.), The SAGE handbook of qualitative research (pp. 796–817). Thousand Oaks, CA: Sage.

**It is also not clear how disagreements between annotators were resolved,
the paper states that "consensus codes where then used" but does not
explain how these were derived and what the impact of having disagreement
is on the descriptive statistics derived from them. Nor is there any analysis
of what the variation of the analysis would be based on the disagreements.**

Upon review, we agree with the reviewer that the term "consensus codes" was confusing. We have revised the sentence to read "the refined codebook" instead of "consensus codes". The previous sentences describe how the refined codebook was derived - from group discussion. We follow similar methodologies published using Twitter data and qualitative approaches such as: Jenkins, E. M., Zaher, Z., Tikkanen, S. A., & Ford, J. L. (2019). Creative identity (re) Construction, creative community building, and creative resistance: A qualitative analysis of queer ingroup members' tweets after the Orlando Shooting. Computers in Human Behavior, 101, 14-21.

**Are annotations seen as a manual attempt to find a latend underlying "true
label assignment" or can disagreements be seen as valid different opinions
at least in some cases? What to do about that for the analysis?**

The reviewer here brings up an important epistemological question. Our team did operate under the premise that disagreements could be valuable in some cases; hence our emphasis on the group discussion rather than statistical methods. We believe that the patterns in the data identified hold true, even accounting for some disagreement.

**The emotion label descriptions in table 1 are rather concise and it is hard to
imagine they would be sufficient for such a complex annotation task, are there
more detailed annotation guidelines and are they part of the data shared?**
We have reviewed our notes from our discussion to add further detail to the codebook. The codebook was designed to hold our notes to ensure the team had an understanding of the codes. Our group discussion was a key part of creating this understanding. The codebook was designed for internal team use, rather than to create a tool that could be used by others. The descriptions were sufficient for our team's agreement.

**Unfortunately the data was not at all available for review, access has been prevented
until 2021-12-15. There is also no metadata which would provdide and exact description of
the data, included field and field values that would allow to judge the usefulness of the data.
There is no information e.g. if all the original label assignments by individual annotators
are included and if assignments made by identical annotators can be identified.
It has to be noted that the submission form for this paper states that
"all data are fully available without restriction" however the data is not available at
all at the time of review and the beginning of the paper states "Data will be made available
upon reasonable request to the authors" which could mean anything including that no request
is accepted as reasonable by the authors.
There have to be doubts therefore that the data can be used in any way to replicate the results
in the paper or go beyond them.**
We have made data available at the link and the restriction is no longer in place.

**The analysis of the manual annotations do not really appear to provide that many new and valuable insights,
when compared to the effort. However, it may be possible to do some further analysis and sharing the
data with the research community in an open and unrestricted way would enable additional
valuable research.**

We have reviewed our discussion section to identify places to clarify the insights provided by this study. We have made our data available such that others can conduct further analyses as desired.

**While the paper is written in clear English it is not well structured and often glosses over details
or presents information in a way where it is hard to understand what has been done exactly.**

We have added additional methodological details to improve the specificity. We have exposed the paper to two more levels of review and changed things such as providing our kappas by code, including details about how coders treated each tweet with options to not code at all, and others. We have added headings in the discussion and reviewed the manuscript for overall flow.

**Reviewer #2: A total of 16,749 tweets between 4/30/2019 and 5/7/2019 were manually analyzed to identify the various emotions following the UNC Charlotte mass shooting and to explore the nature of non-affective short-term reactions to the tragic event. This is an overall well-conducted and interesting study. The temporal trend of annotated categories is informative. The authors did a superb job in the Discussion.**

We appreciate the reviewer's acknowledgement of the usefulness of the temporal trend analysis and the discussion section.

**Major comments:
1. Can the final annotation guideline/codebook be included as a supplement for review? Some of the categories such as Communal Response appear tricky to distinguish from others. Could any same piece of text be labeled with multiple categories?**

The final codebook is what was included in the text. We can include the initial preliminary codebook to show our evolution, if that is desired by reviewers (although this would be atypical for qualitative analyses). We have reviewed the codebook and our notes to augment places from our group discussion. Yes, excerpts could be labeled with multiple categories, as we state on page 8 line 184 "It should be noted that tweets may include several categories"

**2. The separate percentages at the code level (lines 185-187) read a bit confusing in addition to the percentages at the tweet level. How should the code-level relative percentages be interpreted quantitively? As we see there are more negative codes than positive codes, does that mean there was indeed an overall stronger negative sentiment or originally human language has finer semantic granularity for negative expressions? The counting by tweets is easy to follow, but counting by the distinct codes would be affected by whether the denominator makes a meaningful 100%. Please clarify.**

We apologize for the confusion. Our intent including the code level information was to indicate that tweets had more than one code applied to them. We recognize that this was hard to interpret and have since removed this section.  **Minor comments:
1. Might present distribution of the number of categories per tweet to give a feel about how poly-topic are the contents.**

We have included the overall percentage of tweets that were coded with more than one code.

**2. Discussion is long and could be organized into subsections.**

We have added subheadings and shortened the discussion as a whole.

**3. The denominator “available tweets” is unclear in line 217.**

We have removed this sentence. As the reviewer points out it adds confusion and detracts from the overall point of the paragraph.

**4. The relative few worry and fear tweets might also reflect the remote nature of social media. It would be interesting to analyze whether the sentiments varied depending on the distance to the incident (if tractable).**

We agree that this is an interesting question. Given the inconsistent nature of geotagging within the tweets, this is outside of the scope of the current paper.

**5. Could consider color-coding for Figure 1.**

We have added a color-coded Figure.

**Reviewer #3: The main conclusion of this article is that manual coding of tweets provides a more nuanced understanding of reactions to campus mass shootings compared to computational approaches. However, comparing the current findings based on manual annotations to previous studies using computational methods (Jones et al. 2016; De Choudhury et al. 2014) is not the most convincing. It would have been more persuasive if the authors provided computational analysis of exactly the same tweets and showed readers how manual coding is more advantageous. It is also worth noting that both studies on tweeting negative emotions cited here (i.e. Jones et al. 2016; De Choudhury et al. 2014) were published more than 5 years ago. Given how rapidly machine learning and computational linguistics develop in today’s AI era, it is possible that sentiment analysis could be trained to categorize and analyze linguistic information (and emoji) in a more fine-grained way (e.g. Tian et al. 2020; Lekhtman et al. 2021; Suresh & Ong 2021). It would provide a more significant contribution if the authors show the manual coding is more advantageous to the advanced models of computational sentiment analysis.**

We appreciate the reviewer highlighting this oversight. As other reviewers have mentioned, we agree that the argument about manual versus computational methods was problematic. As such, we have removed it from this paper and pivoted our impact to focus more on responses to mass shootings. **It appears that there is no clear cut between some categories. For example, why is the subcategory “blame” a subcategory of “communal response” but not coded as “negative emotions?” Why is “we’ll get through this” coded as “positive emotions” but not under “communal response?” It would be clearer if the authors offer more rigorous criteria for all the categories and subcategories, not simply based on subjective judgments.**
In calling for less of a subjective judgement, the reviewer appears to be preferencing quantitative approaches. Qualitative analyses work toward shared understanding among a team of coders to examine patterns. Our criteria are rigorous in that our team had an understanding of the codebook, even though the excerpts may fall into more than one code.
We agree that there are many nuances to the concepts we're addressing in this work. We have placed blame in communal response due to the inherent social nature of blame and although it includes emotion (such as anger) it is beyond only anger. We based our understanding in part on literature such as: Malle, B. F., Guglielmo, S., & Monroe, A. E. (2014). A theory of blame. Psychological Inquiry, 25(2), 147-186.
In the example provided by the reviewer, "We'll get through this" would be coded with emotion (which is why it is included where it is in the codebook as an example of hope) as well as healing/community response.

**Lastly, some examples may fall into more than one category in the coding system. Would that also be possible for one tweet to fall into more than two categories? I am curious about how the overlapping looks like in terms of statistics. Could the overlapping possibly be visualized? The relevant findings would add more layers to the results and deepen the understanding of how different types of non-affective and/or affective reactions would be more likely to occur in the same tweet or a series of reactions after campus violence.**
We have provided the overall percentage of tweets that were coded with more than one code to help readers understand the nature of the tweets.

**References:
1. Tian, H., Gao, C., Xiao, X., Liu, H., He, B., Wu, H., ... & Wu, F. (2020). SKEP: Sentiment knowledge enhanced pre-training for sentiment analysis. arXiv preprint arXiv:2005.05635
2. Lekhtman, E., Ziser, Y., & Reichart, R. (2021, November). DILBERT: Customized Pre-Training for Domain Adaptation with Category Shift, with an Application to Aspect Extraction. In Proceedings of the 2021 Conference on Empirical Methods in Natural Language Processing (pp. 219-230).
3. Suresh, V., & Ong, D. C. (2021, September). Using Knowledge-Embedded Attention to Augment Pre-trained Language Models for Fine-Grained Emotion Recognition. In 2021 9th International Conference on Affective Computing and Intelligent Interaction (ACII) (pp. 1-8). IEEE.**

**Reviewer #4: In this article, the authors have attempted to perform a content analysis on tweets posted in response to the UNC Charlotte mass shooting event. The authors employed four coders for the manual classification exercise and identified five broad categories: negative affect, positive affect, communal response, action, and information. The authors claim that the study findings reflect the need for university administrators to immediately allay the negative emotions of students in social media. They claim that manual coding is more suitable than computational analysis for Twitter content analysis.

Although the study has certain right justifications, there are multiple issues that need to be addressed before the paper is of publishable quality. The comments are provided below.

1) The authors claim the data has been acquired from public sources. Specific details need to be provided.**

Tweets are publicly available and were gathered using an API. We have added a citation for this. We have added in the keywords that were used, such that our approach could be replicated.

**2) The breakdown from the 75,000 random tweets to the final 16,749 tweets need to be explained more clearly. The sentence “Tweets were divided among four coders and
evaluated for relevancy to UNC Charlotte shooting, Charlotte, NC, national policies, and other incidents of gun violence” means that 75000 was split among four coders. But it doesn’t seem to be the case? Did all the four coders work on the same set of tweets or different. I would recommend rewriting this portion**

We have clarified in this section that the random sample of the 75,000 was divided and evaluated to arrive at the 16,749. The section now reads, "The 75,000 tweets were divided among four coders and evaluated for relevancy to UNC Charlotte shooting, Charlotte, NC, national policies, and other incidents of gun violence. From this, a total of 16,749 tweets fit the criteria in the initial sort."

**3) In multiple instances across the document, the term “sentiment analysis” has been mentioned. However, sentiment polarity is not used in the methods to identify the sentiment of the tweets. Rather, the authors have used emotion classes. Hence, I would suggest refraining the usage of term “sentiment analysis” in this manuscript**

We appreciate the reviewer's clarification. We have added more detail and removed sentiment analysis.

**4) In studies where manual coding is performed, reliability sampling based coding is generally performed as a part of preparation. The authors need to mention details about this exercise if it has been performed. If not performed, are there any justifications?**

We have provided more detail about the type of kappa that was computed and the kappa coefficients have been included in an appendix. As we emphasized a qualitative discussion-oriented approach rather than statistical measures of group understanding, we did not conduct reliability statistics with the full team.

**5) Details need to be provided on how the intercoder agreement was improved. I don’t think it is a good practice to justify separate kappa scores for different codes. Please provide one definite overall kappa score for all the codes. The authors are advised to insert a new table for reporting kappa scores as it is a critical part of the manuscript.**

As aforementioned, we have provided additional information about our reliability calculations. Kappa scores are often provided for different codes in order to identify areas of disagreement, as the goal is clarity in codes so that the coding team can improve the codebook as well as their own understanding of the codes. We have added the table with the kappa scores as an Appendix (as this is often not included with qualitative manuscripts).

**6) I am not sure whether the category codes - negative affect, positive affect, communal response, action, and information are mutually exclusive. The authors need to justify how the latter codes have no affect in the tweets.**

In fact they are not mutually exclusive within the excerpts. An excerpt might be both coded within an affect code as well as action and communal response. However, the codes themselves have been described in such a way that they are exclusive from one another. For instance, in "general action" we have included that this is not policy-specific, individual advocacy or communal response.

**7) The authors need to cite the sources from which the emotion classes were adopted, since there are multiple emotion class schemes put forth in literature**

We have described our approach with literature that formed our basis for the codes, acknowledging that we then adapted from these emotion classes. **8) Lines 184-185 in Page 10 – “total of 50,955 category 185 codes were applied to the tweets”. What does this sentence mean?**

Here we were attempting to describe the number of tweets with multiple codes - we have rephrased this for clarity as: "Most of the tweets (n=14,571, 86.99%) were coded with more than one code." **9) Lines 217-218 in Page 14 – “These categories encapsulated 67.94% (50,955) of available tweets” – What about the remaining tweets?**

We have removed this confusing language, as it was attempting to describe the tweets with multiple codes applied to them.

**10) Sarcasm is not always a positive emotion as the authors have attributed it in the results. In many cases, sarcastic tweets have negative connotations and consequences. Authors could think about this phenomenon and make changes accordingly.**

We agree with the reviewer that sarcasm is hard to categorize - as demonstrated by a wealth of literature focused on natural language processing and sarcasm. We have added a footnote to the table to help explain our use of sarcasm as a positive affect: "Sarcasm usually includes positive affect describing negative situations, and it has been used as a coping mechanism in difficult times to increase well-being (31)".

**11) The implications of this study are not strong in its current form. The authors need to provide more information on how future studies could be benefit from this study.**

We have reviewed the discussion for areas in which we can strengthen and clarify the implications of this study.

**12) The authors claim manual coding is better suited to Twitter analysis than computational approaches. They cannot make this claim without providing evidence. Perhaps, they could do comparison between manually coded tweets and automatically coded tweets for the different emotion classes.**

We have removed the language focused on manual versus computational approaches. Upon review, we agree that other recent studies using computational approaches also provide nuanced approaches. We have shifted our contribution to focus more on the impacts of mass shootings on college campuses.

**13) The coded data should be made available for public access.**

As described above, we have made the data available.
